# Concurrent Dynamics of Category Learning and Metacognitive Judgments

**DOI:** 10.3389/fpsyg.2016.01473

**Published:** 2016-09-27

**Authors:** Valnea Žauhar, Igor Bajšanski, Dražen Domijan

**Affiliations:** Department of Psychology, Faculty of Humanities and Social Sciences, University of RijekaRijeka, Croatia

**Keywords:** categorization, category learning, confidence, dynamics, metacognition

## Abstract

In two experiments, we examined the correspondence between the dynamics of metacognitive judgments and classification accuracy when participants were asked to learn category structures of different levels of complexity, i.e., to learn tasks of types I, II, and III according to Shepard et al. (1961). The stimuli were simple geometrical figures varying in the following three dimensions: color, shape, and size. In Experiment 1, we found moderate positive correlations between confidence and accuracy in task type II and weaker correlation in task type I and III. Moreover, the trend analysis in the backward learning curves revealed that there is a non-linear trend in accuracy for all three task types, but the same trend was observed in confidence for the task type I and II but not for task type III. In Experiment 2, we found that the feeling-of-warmth judgments (FOWs) showed moderate positive correlation with accuracy in all task types. Trend analysis revealed a similar non-linear component in accuracy and metacognitive judgments in task type II and III but not in task type I. Our results suggest that FOWs are a more sensitive measure of the progress of learning than confidence because FOWs capture global knowledge about the category structure, while confidence judgments are given at the level of an individual exemplar.

## Introduction

Metacognitive monitoring of remembering and text comprehension has been studied extensively over recent decades (Dunlosky and Metcalfe, 2009). Less is known about the properties of metacognitive judgments and the availability of different cues in other domains such as category learning. Recently, Jacoby et al. (2010), Wahlheim et al. (2011), Wahlheim et al. (2012) performed a series of studies on metacognition during the learning of natural concepts. They introduced a novel metacognitive measure called category learning judgment (CLJ) that estimates learners’ sensitivity to differences in classification difficulty among categories (families of birds). The results showed that CLJs are higher in conditions that improved classification accuracy for studied and novel exemplars. In particular, CLJs are higher in repeated testing than in repeated study conditions (Jacoby et al., 2010) and in spaced relative to massed study (Wahlheim et al., 2011). Furthermore, Wahlheim et al. (2012) found that CLJs are sensitive to repetitions but not to the variability of exemplars, although both manipulations improved performance. These findings suggest that participants are aware of the beneficial effects of repeated testing and of spaced learning but are also less sensitive to the variability of exemplars. Interestingly, in the context of self-regulated learning, when participants made a choice whether to receive more variability among exemplars from the same category or more repetitions of the same exemplars, they consistently chose to receive more variability (Wahlheim and DeSoto, 2016). However, their study choices were not related to CLJs made after initial exposure to representative exemplars, suggesting that their preferences were based on theoretical beliefs.

In previous studies (Jacoby et al., 2010; Wahlheim et al., 2011, 2012), metacognitive judgments were provided after the learning phase was over and before the transfer phase began. However, it is of equal importance to establish how well the dynamics of metacognitive judgments track the dynamics of category learning. Recently, Doyle and Hourihan (2015) investigated the dynamics of CLJs while participants learned to classify exemplars forming natural categories. They found a gradual increase in CLJs as a function of number of learning blocks. This increase followed the same trend as categorization accuracy. Interestingly, participants exhibited underconfidence for categories with repeated exemplars, while there was no systematic bias for categories with variable exemplars. Doyle and Hourihan (2015) argued that underconfidence arises from the feedback that participants received after each trial. Moreover, they performed fine-grained analysis by computing trial-by-trial differences in performance and CLJs. This analysis revealed that a correct response is more likely to be followed by a correct response rather than an incorrect response. The same analysis for CLJs showed that the largest increase in CLJs occurred when a correct trial followed an incorrect trial, and the largest decrease in CLJs occurred when an incorrect trial followed a correct trial. These findings suggest that, contrary to popular belief, participants learn more from successful trials relative to unsuccessful trials. Moreover, participants are aware of this beneficial effect, implying that CLJs reflect actual learning of category membership.

Although CLJs offer useful insights into learning of natural categories, there are other contexts, such as rule-based category learning, where a different type of metacognitive monitoring is required. In that case, category membership is defined by logical rules that sharply divide exemplars into two mutually exclusive categories. Therefore, there is no variability across categories, and knowledge of one category implies an equal understanding of the other. In a classical study of Shepard et al. (1961), participants were required to classify eight exemplars that vary in three binary dimensions (e.g., color, shape, and size) into two abstract categories. Category structures are defined according to six logical rules of varying complexity. They are labeled type I through type VI. A task type I involves a one-dimensional rule; that is, exemplars are divided into categories based on values on a single dimension, while other dimensions are irrelevant. A task type II is known as an exclusive-or (XOR) logical rule where correct classification depends on the combination of values from two dimensions. Types III, IV, and V require a combination of values in all three dimensions. However, each of these logical rules can be described by a one-dimensional rule supplemented by the exception to the rule. Finally, type VI also requires the integration of information from all three dimensions, but it is not possible to find simple rule-like regularities (Kurtz et al., 2013).

An analysis of error patterns and proportion of correct responses in the data of Shepard et al. (1961) revealed that it is possible to distinguish the four levels of difficulty. In particular, they found that task type I was easiest to learn, followed by task type II and then task type III. There was no difference in learning difficulty between types III, IV, and V. Finally, type VI stands out as the most difficult task. There are several theoretical accounts of the Shepard et al. (1961) data, including implicit exemplar learning (Kruschke, 1992), explicit generation of rules with memorization of occasional exceptions to these rules (Nosofsky et al., 1994b) and construction of mental models (Goodwin and Johnson-Laird, 2011). Finally, there is a possibility that multiple memory systems (explicit and implicit) work in parallel during the learning of such tasks, as proposed by Ashby et al. (1998, 2011) in the COVIS model and by Erickson and Kruschke (1998) in the ATRIUM model.

The aim of the current study is to examine the dynamics of metacognitive monitoring during rule-based category learning, which was not studied thus far. We used two types of metacognitive judgments: confidence and feeling-of-warmth judgments (FOWs). We investigated to what extent these monitoring processes correspond with actual performance during category learning of tasks labeled types I, II, and III according to Shepard et al. (1961). Furthermore, we compared confidence versus FOWs in their ability to track the dynamics of category learning.

In experiment 1, participants gave confidence judgments after each trial during their attempt to learn category structures. Confidence judgments were used to study trial-by-trial monitoring of classification accuracy of each exemplar during learning. In experiment 2, we used the same categorization tasks, but the participants were asked to give FOW judgments. FOW judgments were used to examine monitoring of the acquisition of the rule underlying category structures. More precisely, participants judged how close they feel they are to the acquisition of the appropriate categorization rule after each block of trials. FOW judgments were originally developed by Metcalfe (1986) to examine the dynamics of metacognitive judgments in problem solving. In particular, she examined the cognitive processes that lead to the production of correct or incorrect solutions in problem solving. Metcalfe and Wiebe (1987) showed that FOW judgments increased gradually in the course of solving algebra or non-insight problems, suggesting that they are accessible to metacognitive monitoring, while they did not increase gradually during solving insight problems. Therefore, in solving algebra and non-insight problems, participants reached a solution by the gradual accumulation and combination of partial information (Metcalfe and Wiebe, 1987; Ackerman, 2014).

Rationale for using FOW judgments in experiment 2 is that several theoretical accounts suggest that rule-based category learning involves rule formation and hypothesis testing (Nosofsky et al., 1994b; Ashby and Maddox, 2005, 2010). For example, generating one-dimensional rules and searching for exceptions to this rule as proposed by the RULEX model is a gradual process requiring the integration of several steps (Nosofsky et al., 1994b). We hypothesized that FOW judgments can be applied to rule-based category learning as a measure of metacognitive monitoring of approaching to the acquisition of the appropriate classification rule. Similarly as CLJs, FOWs are measures of metacognitive monitoring at the global category level.

Based on previous work, we expected to find the typical order of classification accuracy with type I > type II > type III (Shepard et al., 1961; Kurtz et al., 2013). In the same way, we expected to find a similar pattern in metacognitive judgments. With respect to the dynamics of learning, we expected that metacognitive judgments would follow a similar trend as classification accuracy (Doyle and Hourihan, 2015). Additionally, we predicted that FOWs would be a more accurate measure of classification performance relative to confidence judgments because FOW judgments are based on more diagnostic cues to classification performance. The reason for this is that FOWs monitor a global level of knowledge about category structure. They are given after participants receive feedback about a complete set of exemplars. Consequently, when FOW is given, participants have more information about each exemplar and their relationships. However, confidence judgments are given before feedback is received on each trial. Confidence is tied to a particular exemplar and might be more sensitive to its incorrect category representation.

## Experiment 1

### Method

#### Participants

Forty-four undergraduate psychology students from University of Rijeka, Croatia, participated in the study in exchange for course credits. All participants were tested individually in a quiet, dimly lit room. One participant was removed from the analysis because (s)he failed to learn task type I indicating the lack of motivation to follow the instructions.

#### Materials

The participants learned three classification tasks labeled type I, II, and III by Shepard et al. (1961). The stimuli were eight geometrical figures varying along three binary dimensions: shape (triangle/square), size (small/large), and color (black/white). The underlying rule for task type I was one-dimensional simple logical rule based on color (e.g., category A consisted of black geometrical figures); the conjunctive two-dimensional rule based on color and shape defined task type II (e.g., category A consisted of black triangles *AND* white squares); and the complex three-dimensional rule defined task type III (e.g., category A consisted of black geometrical figures *with the exception* of the small black square *and including* the small white triangle). We did not include task type IV and V because previous work suggests that they are of comparable difficulty to task type III. Additionally, we excluded task type VI because it is the most difficult task and it requires the rote memorization of exemplars. In other words, it is not possible to verbalize a simple logical rule that can solve task type VI.

#### Procedure

Every trial started with the presentation of the stimulus in the center of the screen. The stimulus remained on the screen until the participant made a response. Each of the eight stimuli (geometrical figures) was presented once during a single block or trial in a randomized order. Participants were instructed to determine whether the presented stimulus (geometrical figure) belonged to category A or B by pressing appropriate keys on the computer keyboard. If they were not sure how to classify an exemplar, they were instructed to guess its category. After each classification, participants were asked to give confidence judgments on a scale ranging from 50 (*‘not confident at all’*) to 100% (*‘completely confident’*) by pressing appropriate keys on the computer keyboard. Each rating was followed by feedback on classification accuracy. Learning continued until participants reached 16 consecutive correct classifications within two blocks or a maximum of 20 blocks (160 trials). Upon completion of the classification task, subjects were asked to write the underlying rule on a sheet of paper. The same procedure was repeated for all tasks. Participants completed all three learning tasks. The order of presentation of tasks was randomized across participants.

## Results and Discussion

Data were analyzed on three different levels. Firstly, we analyzed mean performance when data are aggregated across all learning blocks. Secondly, we analyzed trends in backward learning curves. In addition, we performed trial-by-trial analysis similar to Doyle and Hourihan (2015) that is presented in Supplemental Material.

Due to substantial departures from sphericity, data were analyzed using MANOVAs instead of ANOVAs with sphericity corrections. We reported the results of the multivariate Pillai test. In the same manner, the Pillai test was applied in *post hoc* comparisons and trend analyses with Holm adjustment of the *p* values as a protection against α-error. In all analyses, the significance level was set at 0.05.

### Mean Performance

We analyzed the number of learning blocks, accuracy, confidence judgments, and log_10_ transformed response times using four separate one-way MANOVAs with tasks (I, II, or III) as a repeated measure factor. If a participant achieved maximal accuracy in two consecutive blocks before the end of the session, we assumed that (s)he would continue to do so in all subsequent blocks and filled the empty cells accordingly (Nosofsky et al., 1994a). In the same manner, we assumed that confidence would be maximal in all subsequent blocks.

#### Number of Learning Blocks

One-way MANOVA (*N* = 43) revealed a significant main effect of the task, [*F*(2,41) = 130.60, *p* < 0.001, $\eta_{p}^{2}$ = 0.86]. Task type I was learned faster (*M* = 4.16, *SE* = 0.30) than task type II (*M* = 10.58, *SE* = 0.92), *F*(1,42) = 43.00, *p* < 0.001, $\eta_{p}^{2}$ = 0.51, or task type III (*M* = 15.86, *SE* = 0.74), *F*(1,42) = 260.39, *p* < 0.001, $\eta_{p}^{2}$ = 0.86. Additionally, task type II was learned faster than task type III, *F*(1,42) = 24.54, *p* < 0.001, $\eta_{p}^{2}$ = 0.37.

#### Categorization Accuracy

One-way MANOVA (*N* = 43) revealed a significant main effect of the task, *F*(2,41) = 107.59, *p* < 0.001, $\eta_{p}^{2}$ = 0.84. Accuracy was higher in task type I (*M* = 97.37%, *SE* = 0.54) than in task type II (*M* = 81.92%, *SE* = 2.39), *F*(1,42) = 41.35, *p* < 0.001, $\eta_{p}^{2}$ = 0.50, or task type III (*M* = 75.16%, *SE* = 1.73), *F*(1,42) = 184.50, *p* < 0.001, $\eta_{p}^{2}$ = 0.81. Additionally, accuracy was higher in task type II than III, *F*(1,42) = 5.60, *p* = 0.023, $\eta_{p}^{2}$ = 0.12. This is consistent with the order of tasks according to their difficulty (i.e., I > II > III) as observed in previous studies (Shepard et al., 1961; Kurtz et al., 2013).

#### Confidence Judgments

One-way MANOVA (*N* = 43) revealed a significant main effect of the task, *F*(2,41) = 62.33, *p* < 0.001, $\eta_{p}^{2}$ = 0.75. Confidence was higher in task type I (*M* = 97.25%, *SE* = 0.55) than in task type II (*M* = 86.11%, *SE* = 1.98), *F*(1,42) = 36.32, *p* < 0.001, $\eta_{p}^{2}$ = 0.46, or task type III (*M* = 81.77%, *SE* = 1.54), *F*(1,42) = 113.82, *p* < 0.001, $\eta_{p}^{2}$ = 0.73. Furthermore, confidence was higher in task type II than III, *F*(1,42) = 4.36, *p* = 0.043, $\eta_{p}^{2}$ = 0.09. This analysis confirms our hypothesis that confidence follows the same pattern (i.e., I > II > III) as observed for accuracy.

#### Response Times

One-way MANOVA (*N* = 43) revealed a significant main effect of the task, *F*(2,41) = 80.17, *p* < 0.001, $\eta_{p}^{2}$ = 0.80. Responses were faster in task type I (*M* = 3.022, *SE* = 0.021) than in task type II (*M* = 3.300, *SE* = 0.022), *F*(1,42) = 138.72, *p* < 0.001, $\eta_{p}^{2}$ = 0.77, or task type III (*M* = 3.293, *SE* = 0.018), *F*(1,42) = 118.23, *p* < 0.001, $\eta_{p}^{2}$ = 0.74. Furthermore, there was no statistically significant difference in response times between task types II and III, *F*(1,42) < 1, *p* > 0.50, $\eta_{p}^{2}$ < 0.01. Therefore, response times were not able to distinguish between task types II and III, although there are reliable differences between them observed in accuracy and confidence judgments.

### Backward Learning Curves

In order to investigate dynamics of category learning and metacognitive judgments we constructed backward learning curves (BLCs). This method of analysis solves the problem of individual differences in learning that occur when averaging data within blocks (Hayes, 1953; Smith and Ell, 2015). We took into account last five learning blocks prior to the termination of the learning session and aligned data of each participant accordingly. The last block of all participants was labeled as Block 0, the block that immediately precedes the last block was labeled as Block -1 and so forth. Next, we checked for existence of linear and non-linear trends in BLCs averaged across participants. Of particular interest was the question whether metacognitive judgments follow the same trend as accuracy.

#### Trends in BLCs

Several participants achieved maximal accuracy in less than five blocks. For these participants, we filled missing cells by the corresponding results observed in the last block available. We analyzed accuracy and confidence using three 5 × 3 MANOVAs with the block (-4, -3, -2, -1, 0) and the task (I, II, III) as within-participant factors.

#### Categorization Accuracy

The 5 × 3 MANOVA (*N* = 43) revealed a significant main effect of the block, *F*(4,39) = 64.84, *p* < 0.001, $\eta_{p}^{2}$ = 0.87, and the main effect of the task, *F*(2,41) = 12.21, *p* < 0.001, $\eta_{p}^{2}$ = 0.37. The block × task interaction was also significant, *F*(8,35) = 2.76, *p =* 0.018, $\eta_{p}^{2}$ = 0.39. To examine trends in categorization accuracy, we computed polynomial orthogonal contrasts over the means in BLCs. The results of the trend analysis are displayed in Supplementary Table S2.

**FIGURE 1:**
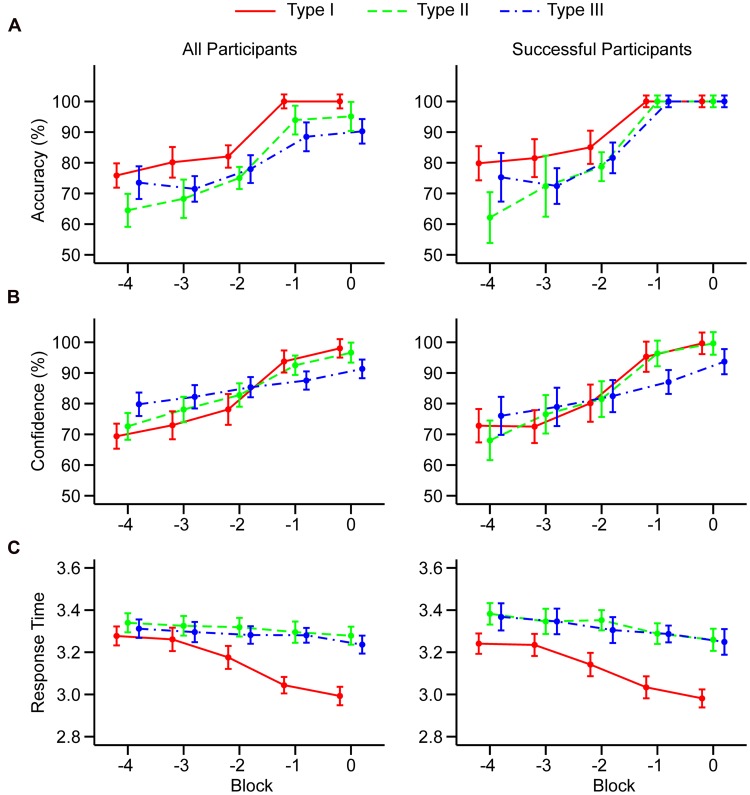
**Mean accuracy (A), confidence judgments (B), and log_10_ transformed response times (C) in the last five learning blocks.** Results are shown separately for all participants (left column) and for participants who successfully learned all three tasks (right column). Blocks are counted relative to the end of the learning session. Error bars represent 95% within-subjects confidence intervals computed following Cousineau (2005) and Morey (2008).

Polynomial contrasts revealed that there was a statistically significant linear component in all task types. There was no significant quadratic component, but there was a significant cubic component in all task types. These results suggest that classification accuracy showed a trend with a steep increase in Block -2 that reached a plateau over Blocks -1 and 0, as shown in **Figure 1**. Importantly, a similar trend was observed for all three task types. In addition, there was quartic trend in task types I and II.

#### Confidence Judgments

The 5 × 3 MANOVA (*N* = 43) revealed a significant main effect of the block, *F*(4,39) = 41.45, *p* < 0.001, $\eta_{p}^{2}$ = 0.81, but there was no significant main effect of the task, *F*(2,41) = 0.72, *p* = 0.491, $\eta_{p}^{2}$ = 0.03. The block × task interaction was significant, *F*(8,35) = 4.96, *p* < 0.001, $\eta_{p}^{2}$ = 0.53. Trend analysis revealed that there was a statistically significant linear component in all three task types. However, in the task type I, there was also a significant cubic trend similar to that observed in accuracy, but there were no significant non-linear trends for task types II and III. This analysis suggests that confidence judgments for task type I follow a non-linear sigmoid-like trend similar to that observed in accuracy. However, confidence in task type II and III follow a slower linear trend, suggesting that participants are more cautious and that they are not yet willing to give maximal confidence judgment in the block where they achieved maximal accuracy.

#### Response Times

The 5 × 3 MANOVA (*N* = 43) revealed a significant main effect of the block, *F*(4,39) = 13.99, *p* < 0.001, $\eta_{p}^{2}$ = 0.59, and the main effect of the task, *F*(2,41) = 17.04, *p* < 0.001, $\eta_{p}^{2}$ = 0.45. The block × task interaction was also significant, *F*(8,35) = 5.64, *p* < 0.001, $\eta_{p}^{2}$ = 0.56. **Table 1** showed that there is a significant linear and cubic trend for task type I, suggesting that the response became faster as participants reached the end of the learning session. However, there was no evidence for any type of trend in task types II and III because they were much more difficult to learn and their response times were not indicative of performance, as shown by the correlational analysis.

**Table 1:** Correlations between performance measures observed in Experiment 1.

Learning Task						
	**Type I**		**Type II**		**Type III**	
	**RT**	**CONF**	**RT**	**CONF**	**RT**	**CONF**
ACC	-0.35	0.25	0.36*	0.41*	0.06	0.27
RT		-0.32		-0.11		0.09

*p < 0.05. Statistical significance was assessed by oxt-test with 41° of freedom and Holm correction for multiple comparisons. NB, number of blocks; ACC, accuracy; RT, response times; CONF, confidence.

#### Trends in BLCs with Successful Participants

Participants who failed to learn one of the tasks introduce additional noise in the data that might obscure existing trends or artificially create non-existing trends. To address this problem, we separately analyzed the dataset restricted to participants who successfully learned all three tasks. Twenty-one participants were removed from the analysis. It should be noted that this analysis cannot be performed on accuracy due to the lack of variability in Blocks -1 and 0. However, visual inspection of **Figure 1A** (right column) showed that non-linear sigmoid-like trend in accuracy is present in all three task types.

#### Confidence Judgments

The 5 × 3 MANOVA (*N* = 22) revealed a significant main effect of the block, *F*(4,18) = 45.29, *p* < 0.001, $\eta_{p}^{2}$ = 0.91, although there was no main effect of the task, *F*(2,20) < 1, *p* > 0.50, $\eta_{p}^{2}$ < 0.01. The block × task interaction was significant, *F*(8,14) = 5.01, *p* = 0.004, $\eta_{p}^{2}$ = 0.74. Trend analysis (Supplementary Table S3) showed that linear trends remained significant in all task types. Also, in task type I, cubic trend remained significant although quartic trend disappeared. Furthermore, there appeared evidence for quartic trend in task type II. All other trends remained non-significant.

#### Response Times

The 5 × 3 MANOVA (*N* = 22) revealed a significant main effect of the block, *F*(4,18) = 9.17, *p* < 0.001, $\eta_{p}^{2}$ = 0.67, and a significant main effect of the task, *F*(2,20) = 29.62, *p* < 0.001, $\eta_{p}^{2}$ = 0.75. The block × task interaction was marginally significant, *F*(8,14) = 2.64, *p =* 0.054, $\eta_{p}^{2}$ = 0.60. Linear and cubic trends remained significant in task type I. All other trends remained non-significant (Supplementary Table S3).

This analysis showed that non-linear trends (cubic or quartic) in confidence exist in the task type I and II although similar non-linear trends in accuracy is observed in all three learning tasks. Also, non-linear component in response times was observed in the task type I suggesting sharp increase in speed of response as participants approached criterion.

#### Correlations between Performance Measures

**Table 1** displays Pearson’s correlation coefficients that were computed for all performance measures when data are aggregated across BLC blocks. There are no statistically significant correlations between performance measures in task type I and III. On the other hand, classification accuracy is positively correlated with confidence judgments in the task type II. Lack of accuracy-confidence correlation in the task type I might be due to the restricted range in accuracy because all participants learned this task.

## Experiment 2

Experiment 1 revealed that the dynamics of confidence closely corresponds with the dynamics of accuracy in task type I and II but not in task type III. This finding suggests that confidence judgments are less accurate in monitoring more complex tasks that require a combination of information from three dimensions. In Experiment 2, we sought to establish whether different types of metacognitive judgment such as FOW might be able to track the dynamics of category learning in task type III.

### Method

#### Participants

Thirty-eight undergraduate psychology students from the University of Rijeka, Croatia, participated in an exchange for course credits. All participants were tested individually. Three participants were removed from the analysis due to the equipment failure during the experimental session.

#### Materials

The stimuli and classification tasks were identical to those used in Experiment 1.

#### Procedure

Category learning tasks were applied in the same way as in Experiment 1, except that the participants were not required to make confidence judgments after each response. Instead, participants provided FOW judgments to assess their subjective feeling of how close they believe they were to the discovery of an appropriate classification rule. They were asked to indicate their FOW judgments after each complete block of eight trials on a 7-point scale ranging from 1 (meaning *‘not close at all to the appropriate classification rule’*) to 7 (meaning *‘completely confident about the appropriate classification rule’*). In addition, after FOW judgments, participants were asked to write down the underlying rule or to describe features characterizing categories A and B on a sheet of paper.

## Results and Discussion

### Mean Performance

Analysis was performed in the same way as in Experiment 1. We analyzed the number of learning blocks, accuracy, FOW judgments and log_10_ transformed response times in four separate MANOVAs with the task (I, II, III) as a repeated measure factor.

#### Number of Learning Blocks

One-way MANOVA revealed a significant main effect of a category learning task, *F*(2,33) = 109.40, *p* < 0.001, $\eta_{p}^{2}$ = 0.87. The number of blocks was smaller in task type I (*M* = 4.43, *SE* = 0.37) than task type II (*M* = 11.29, *SE* = 1.00), *F*(1,34) = 57.43, *p* < 0.001, $\eta_{p}^{2}$ = 0.63, and task type III (*M* = 15.71, *SE* = 0.74), *F*(1,34) = 202.86, *p* < 0.001, $\eta_{p}^{2}$ = 0.86. Furthermore, the number of blocks was smaller in task type II than III, *F*(1,34) = 17.03, *p* < 0.001, $\eta_{p}^{2}$ = 0.33.

#### Categorization Accuracy

One-way MANOVA (*N* = 35) revealed a significant main effect of the task, *F*(2,33) = 73.95, *p* < 0.001, $\eta_{p}^{2}$ = 0.82. Accuracy was higher in task type I (*M* = 97.23%, *SE* = 0.66) than task type II (*M* = 79.04%, *SE* = 2.78), *F*(1,34) = 51.50, *p* < 0.001, $\eta_{p}^{2}$ = 0.60, and task type III (*M* = 71.00%, *SE* = 2.50), *F*(1,34) = 125.42, *p* < 0.001, $\eta_{p}^{2}$ = 0.79. Furthermore, accuracy was higher in task type II than III, *F*(1,34) = 6.65, *p* = 0.014, $\eta_{p}^{2}$ = 0.16. This confirms the typical pattern (I > II > III) observed in previous studies and in Experiment 1.

#### FOW Judgments

One-way MANOVA (*N* = 35) revealed a significant main effect of the task, *F*(2,33) = 92.27, *p* < 0.001, $\eta_{p}^{2}$ = 0.85. FOW was higher in task type I (*M* = 6.79, *SE* = 0.05) than task type II (*M* = 5.04, *SE* = 0.28), *F*(1,34) = 45.12, *p* < 0.001, $\eta_{p}^{2}$ = 0.57, and task type III (*M* = 3.90, *SE* = 0.23), *F*(1,34) = 173.86, *p* < 0.001, $\eta_{p}^{2}$ = 0.84. Furthermore, FOW was higher in task type II than III, *F*(1,34) = 14.17, *p* < 0.001, $\eta_{p}^{2}$ = 0.29. This confirms our hypothesis that FOW judgments follow the same pattern as accuracy and that they are sensitive to the task difficulty.

#### Response Times

One-way MANOVA (*N* = 35) revealed a significant main effect of the task, *F*(2,33) = 74.33, *p* < 0.001, $\eta_{p}^{2}$ = 0.82. Responses were faster in task type I (*M* = 2.888, *SE* = 0.028) relative to task type II (*M* = 3.209, *SE* = 0.030), *F*(1,34) = 152.41, *p* < 0.001, $\eta_{p}^{2}$ = 0.82, or task type III (*M* = 3.184, *SE* = 0.027), *F*(1,34) = 73.91, *p* < 0.001, $\eta_{p}^{2}$ = 0.68. Furthermore, there was no statistically significant difference in response times between task types II and III, *F*(1,34) < 1, *p* = 0.346, $\eta_{p}^{2}$ = 0.03. As in Experiment 1, response times did not distinguish between task types II and III, although there were clear differences between them observed in accuracy and FOWs.

### Backward Learning Curves

As in Experiment 1, we constructed BLCs and analyzed trends in accuracy and FOWs as a function of learning block.

#### Trends in BLCs

**FIGURE 2:**
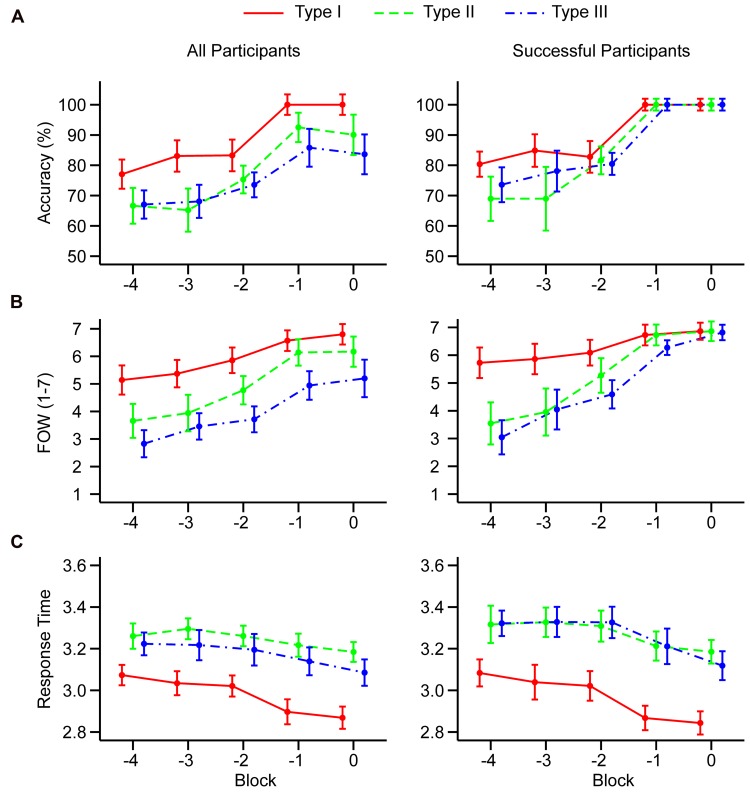
**Mean accuracy (A), feeling-of-warmth judgments (B), and log_10_ transformed response times (C) in the last five learning blocks.** Results are shown separately for all participants (left column) and for participants who successfully learned all three tasks (right column). Blocks are counted relative to the end of the learning session. Error bars represent 95% within-subjects confidence intervals computed following Cousineau (2005) and Morey (2008).

We analyzed accuracy and FOW judgments in the BLCs using three separate 5 × 3 MANOVAs with the block (-4, -3, -2, -1, 0) and the task (I, II, III) as within-participant factors. Means and within-subjects confidence intervals across all conditions are plotted in **Figure 2**.

#### Categorization Accuracy

The 5 × 3 MANOVA (*N* = 35) revealed a significant main effect of the block, *F*(4,31) = 33.97, *p* < 0.001, $\eta_{p}^{2}$ = 0.81, and a significant main effect of the task, *F*(2,33) = 11.67, *p* < 0.001, $\eta_{p}^{2}$ = 0.41. The block × task interaction was not significant, *F*(8,27) = 1.98, *p =* 0.089, $\eta_{p}^{2}$ = 0.40. Trend analysis revealed a significant linear component in all three tasks (Supplementary Table S4). Furthermore, task type I showed a significant cubic and quartic component, while task types II and III showed a significant cubic component. Replicating the pattern from Experiment 1, trend analysis suggests that in all task types, there is evidence of a non-linear trend near the end of learning.

#### FOW Judgments

The 5 × 3 MANOVA (*N* = 35) revealed a significant main effect of the block, *F*(4,31) = 19.51, *p* < 0.001, $\eta_{p}^{2}$ = 0.72, and a significant main effect of the task, *F*(2,33) = 29.28, *p* < 0.001, $\eta_{p}^{2}$ = 0.64. The block × task interaction was not significant, *F*(8,27) = 1.69, *p =* 0.148, $\eta_{p}^{2}$ = 0.33. Trend analysis revealed statistically significant linear component, and lack of quadratic component in all three task types (Supplementary Table S4). In addition, there was a significant cubic component in task type II. There was no evidence for quartic trend in neither of task types.

#### Response Times

The 5 × 3 MANOVA (*N* = 35) revealed a significant main effect of the block, *F*(4,31) = 11.18, *p* < 0.001, $\eta_{p}^{2}$ = 0.59, and a significant main effect of the task, *F*(2,33) = 50.69, *p* < 0.001, $\eta_{p}^{2}$ = 0.75. The block × task interaction was not significant, *F*(8,27) = 1.89, *p =* 0.104, $\eta_{p}^{2}$ = 0.36. Trend analysis revealed that there is a significant linear trend in task type I and III. Also, there is a quartic trend in task type I. All other trends were non-significant.

#### Trends in BLCs with Successful Participants

As in Experiment 1, we performed separate analysis on the dataset restricted to participants who successfully learned all three tasks (Supplementary Table S5). Thirteen participants were removed from the analysis. Again, this analysis could not be performed on accuracy due to the lack of variability in Blocks -1 and 0. However, visual inspection of **Figure 2A** (right column) showed that non-linear sigmoid-like trend in accuracy is present in all three task types because there is an abrupt rise in accuracy between Blocks -2 and -1.

#### FOW Judgments

The 5 × 3 MANOVA (*N* = 22) revealed a significant main effect of the block, *F*(4,18) = 32.71, *p* < 0.001, $\eta_{p}^{2}$ = 0.88, and a significant main effect of the task, *F*(2,20) = 11.63, *p* < 0.001, $\eta_{p}^{2}$ = 0.54. The block × task interaction was also significant, *F*(8,14) = 7.16, *p* < 0.001, $\eta_{p}^{2}$ = 0.80. Linear trend remained significant in all three task types. In task type II, cubic trend also remained significant. Furthermore, a significant quartic trend emerged in task type III. All other trends remained non-significant.

#### Response Times

The 5 × 3 MANOVA (*N* = 22) revealed a significant main effect of the block, *F*(4,18) = 14.35, *p* < 0.001, $\eta_{p}^{2}$ = 0.76, and a significant main effect of the task, *F*(2,20) = 36.70, *p* < 0.001, $\eta_{p}^{2}$ = 0.79. The block × task interaction was not significant, *F*(8,14) = 1.47, *p =* 0.252, $\eta_{p}^{2}$ = 0.46. Linear trend in task type I and III remained significant as well as quartic trend in task type I. All other trends remained non-significant.

The analysis suggests that the dynamics of FOW judgments is well aligned with the dynamics of accuracy in task type II and III but not in task type I. The lack of non-linear trend in task type I is related to the fact that FOW judgments are already high at Block -4 and there is no much opportunity to exhibit non-linear transition to maximal response. However, non-linear trend in task type I is observed in response times that exhibited sharp reduction between Blocks -2 and -1.

#### Correlations between Performance Measures

**Table 2** displays Pearson’s correlation coefficients, calculated for all performance measures in all tasks when data are aggregated across BLC blocks. There is a moderate negative correlation between RT and accuracy and between RT and FOW judgments in task type I. On the other hand, these correlations are positive in task type III suggesting qualitative differences between the tasks. Task type I is easy to learn and when participants identify single relevant dimension, they become faster, more accurate and give higher FOW judgments. Task type III requires more cognitive effort that is reflected in slower response times for participants who learned it and who also gave higher FOW judgments. Importantly, correlation between accuracy and FOW judgments was positive and high in all task types. It is interesting to note that, when compared to accuracy-confidence correlations observed in Experiment 1, the associations between accuracy and FOW judgment in Experiment 2 are generally stronger. Below, we provide quantitative assessment of this difference in the separate section Comparison between Experiments.

**Table 2:** Correlations between performance measures observed in Experiment 2.

Learning Task						
	**Type I**		**Type II**		**Type III**	
	**RT**	**FOW**	**RT**	**FOW**	**RT**	**FOW**
ACC	-0.35*	0.50*	0.16	0.74*	0.48*	0.68*
RT	-0.43*		-0.03	0.37*

*p < 0.05. Statistical significance was assessed by oxt-test with 33° of freedom and Holm correction for multiple comparisons. NB, number of blocks; ACC, accuracy; RT, response times; FOW, feeling-of-warmth judgments.

### Comparison between Experiments

Although suggestive, qualitative differences between trends in BLCs provide only indirect evidence that FOW judgments follow dynamics of learning more closely than confidence judgments. In order to provide more direct test, we computed Fisher’s *z*-test for the comparison between two Pearson’s coefficients of correlation found in two independent samples. With Fisher’s *z*-test, we compared the size of the accuracy-confidence correlation (*r_1_*) found in Experiment 1 with the size of accuracy-FOW correlation (*r_2_*) found in Experiment 2. Also, we computed 95% confidence intervals around the difference between correlations as proposed by Zou (2007).

In task type I, difference between correlation coefficients (*r_1_* = 0.25 vs. *r_2_* = 0.50) was not significant, *d* = -0.25, 95% CI = [-0.62, 0.15], *z* = -1.24, *p* = 0.215. However, in task type II, accuracy-confidence correlation was significantly lower than accuracy-FOW correlation (*r_1_* = 0.41 vs. *r_2_* = 0.74), *d* = -0.33, 95% CI = [-0.64, -0.03], *z* = -2.17, *p* = 0.030. In task type III, again accuracy-confidence correlation was significantly lower than accuracy-FOW correlation (*r_1_* = 0.27 vs. *r_2_* = 0.68), *d* = -0.41, 95% CI = [-0.75, -0.06], *z* = -2.33, *p* = 0.020. These comparisons suggest that FOW judgments are more sensitive than confidence to classification accuracy in the task types II and III. It is interesting to note that these are the same tasks where non-linear trends are observed in FOW judgments.

Furthermore, we examined the differences between accuracy-confidence and accuracy-FOW correlations across blocks in the BLCs. Results of these comparisons are displayed in **Table 3**. In task type I, there was a significant difference between correlations in Block -4 only. As already noted, task type I might be too easy to learn and there is not enough opportunity for a difference between confidence and FOW to emerge. Also, it should be noted that it was not possible to compute correlation in Block -1 and 0 because there was no variability in accuracy. More importantly, in task type II, significantly higher accuracy-judgment correlations are observed in the last three blocks, that is, near the end of learning. The same pattern was also observed in the task type III. Differences between correlations in Blocks -2, -1, and 0 suggest that FOW judgments are better aligned with changes in accuracy that occurred when participants learned the task. Therefore, accuracy-FOW correlations are higher than accuracy-confidence correlations because FOW judgments capture the moment when the task is learned. On the other hand, it seems that confidence judgments require additional positive feedback in the last blocks and consequently fail to align with accuracy.

**Table 3:** Differences between the accuracy-judgment correlations observed in Experiment 1 and 2 across learning blocks.

Task	Block	*r_1_* (41)	*r_2_* (33)	*d*	95% CI	*z*	*p*
Type I	-4	0.12	0.62	-**0.50**	[0.85, -0.11]	-2.55	0.011
	-3	0.43	0.64	-0.21	[-0.53, 0.12]	-1.26	0.209
	-2	0.30	0.53	-0.23	[-0.59, 0.15]	-1.18	0.237
	-1	–	–	–	–	–	–
	0	–	–	–	–	–	–
Type II	-4	0.37	0.63	-0.26	[-0.60, 0.09]	-1.49	0.137
	-3	0.42	0.42	0	[-0.37, 0.39]	<0.10	>0.50
	-2	0.30	0.66	**-0.36**	[-0.70, -0.01]	-2.04	0.042
	-1	0.42	0.78	**-0.36**	[-0.66, -0.08]	-2.52	0.012
	0	0.62	0.90	**-0.28**	[-0.51, -0.10]	-3.15	0.002
Type III	-4	0.37	0.40	-0.03	[-0.41, 0.37]	-0.15	>0.50
	-3	0.54	0.60	-0.06	[-0.37, 0.26]	-0.38	>0.50
	-2	0.17	0.58	**-0.41**	[-0.77, -0.02]	-2.07	0.039
	-1	0.13	0.65	**-0.52**	[-0.87, -0.14]	-2.72	0.007
	0	0.29	0.86	**-0.57**	[-0.88, -0.29]	-4.19	<0.001

*Significant effects are highlighted by bold typeface. r_1_(r_2_), accuracy-judgment correlation observed in Experiment 1 (2); d, difference between r_1_ and r_2_; CI, confidence interval around the difference (following Zou, 2007); z, Fisher’s z-test.*

## General Discussion

In the present study, we employed two distinct metacognitive judgments that addressed category learning from different perspectives. In Experiment 1, we used confidence judgments after the classification of each exemplar. In this condition, participants are encouraged to think about each exemplar as an isolated item without considering its relationship with other items. In Experiment 2, participants gave FOW judgments after a block of trials. In this case, participants are encouraged to think about exemplars as a group of items that are united by a rule. An analysis of the mean performance showed that in both experiments, accuracy followed the same pattern, I > II > III, that is commonly observed in studies using the tasks introduced by Shepard et al. (1961). Confidence and FOW judgments also showed the same pattern, suggesting that both metacognitive measures are sensitive to task difficulty. On average, confidence judgments were higher than actual performance in task type II and III but not in task type I, where almost perfect alignment between confidence and accuracy was achieved. This is not surprising because task type I is easy to master by identifying a single relevant dimension, which is also easy to monitor.

We studied dynamics of category learning and metacognitive judgments by constructing BLCs and analyzing their trends. This analysis revealed a dissociation between the dynamics of classification accuracy and confidence judgments in Experiment 1. In particular, we computed polynomial orthogonal contrasts to show that classification accuracy follows a non-linear trend in all three task types. However, confidence judgments follow a non-linear trend only in task type I and II, while task type III follows a linear trend. It seems that participants were more cautious in judging their performance in task type III near the end of learning. The reason for this caution is the fact that in the starting block, participants greatly overestimated their performance. Massive negative feedback due to the large number of error forced them to reconsider their estimates in later blocks.

Trend analysis in Experiment 2 showed that accuracy and FOW judgments followed non-linear trends in task type II and III but not in task type I. A non-linear trend in task type III suggests that FOW judgments more accurately tracks classification performance near the end of learning relative to confidence in this most difficult task. In particular, FOW judgments better capture the transition to perfect performance because they reached a plateau in the last two blocks in the similar way as classification accuracy did. On the other hand, it seems that FOW judgments are not particularly suited for tracking performance in easy task such as type I.

We also examined correlations between accuracy and metacognitive judgments. In general, accuracy-FOW correlations (*r_2_*) observed in Experiment 2 were higher relative to accuracy-confidence correlations (*r_1_*) observed in Experiment 1 across all task types. When we directly compared the size of correlations in Experiment 1 and 2, we found that *r_2_* were significantly higher than *r_1_* in task types II and III. Moreover, when correlations were compared across blocks in BLCs, we found that association between accuracy and FOW is stronger than association between accuracy and confidence in Blocks -2, -1 and 0, that is, near the end of learning. Such finding suggests that FOW judgments are more sensitive to transition in accuracy that occurred when participants approached criterion level of performance. In other words, FOWs are a better measure of success in category learning.

Comparisons between accuracy-confidence and accuracy-FOW correlations also corroborate trend analysis in BLCs because advantage of FOWs over confidence is seen in the same tasks where FOW judgments exhibited non-linear trends similar to those observed in accuracy. Therefore, correlations and trends in BLCs provide complementary view on the dynamics of category learning and metacognitive judgments. On the other hand, lack of difference between *r_1_* and *r_2_* as well as inconsistencies between correlational and trend analysis found in task type I^1^ can be explained by the ceiling effect, that is, accuracy in both experiments and FOW judgments in the Experiment 2 exhibited low variability in this task. In other words, task type I is easy to learn and participants are aware of this fact irrespective of the type of judgment they provided.

What is the explanation for better tracking of classification performance by FOW judgments compared to confidence judgments? Following the cue-utilization view of metacognitive monitoring (Koriat, 1997), the crucial question is on which cues or sources of information these two types of judgments are based. Many findings in the domain of metamemory show that differences in the accuracy of metacognitive judgments (their correlation with memory performance) depend on the validity of those cues (for example, a delayed JOL effect, Dunlosky and Nelson, 1992, 1994; see also: Koriat, 1997, 2007). Accordingly, the discrepancy between FOWs and confidence given during category learning observed in this study should also arise from the validity of cues on which these judgments are based.

Confidence judgments are given for each particular item before feedback. Therefore, incorrectly formed hypotheses about the category membership of that item should result in a discrepancy between classification accuracy and confidence. Confidence judgments primarily reflect the memory for category membership of a particular exemplar based on experience with previous correct and incorrect classifications of that item.

However, FOWs are based on immediate feedback about the classification accuracy of all eight items in the previous learning block. To the extent that the number of correct and incorrect classifications reflect the level of acquisition of the appropriate rule, it is expected that the FOWs will also be in line with the degree of acquisition of the rule. Therefore, FOWs monitor integrated knowledge about category structure. They are a form of summary representation of the category similar to the CLJs. Thus, FOWs are based on cues that are more diagnostic for actual classification performance.

Alternative explanation of better alignment of FOWs with classification accuracy is that FOWs possess unique features that sets them apart from other types of judgments either global or local. In particular, FOWs are prospective judgments that encourage participants to think about the rule formation and hypothesis testing. Future studies should address the question to what degree are the results of Experiment 2 specific to FOWs. In other words, could these results be generalized to other types of global metacognitive judgments? To this end, a new metacognitive judgment should be devised where participants are asked to assess the level of acquired knowledge after each block of trials, that is, to give a form of global judgment about their knowledge of category structure. In the case that our global/local interpretation is correct, we would expect that category knowledge judgments will exhibit similar dynamics as it was observed with FOWs in Experiment 2. On the other hand, if FOWs are indeed unique metacognitive judgments, we would expect that category knowledge judgments will exhibit similar dynamics as it was found for averaged local confidence in Experiment 1.

Previous studies on the metacognitive monitoring of category learning employed CLJs (Jacoby et al., 2010; Wahlheim et al., 2011, 2012; Doyle and Hourihan, 2015; Wahlheim and DeSoto, 2016). The CLJs estimate the probability of correct classification of novel exemplars from the same category. When participants learned natural categories, it was found that CLJs were sensitive to the repetition of items during study but not sensitive to the variability of exemplars within the categories, although both factors affected actual performance. Furthermore, it was found that the dynamics of CLJs closely follow the progress of category learning (Doyle and Hourihan, 2015). Our study complements these findings by showing that, in the tasks where category membership is defined by logical rules, confidence and FOW judgments are also useful measures of the awareness of the progress of learning. Moreover, we found that FOW judgments better capture the dynamics of accuracy than confidence.

## Author Contributions

DD designed the study, and participated in data analysis and writing of report. VŽ designed the study, and participated in data analysis and writing of report. IB participated in data analysis and writing of report.

## Conflict of Interest Statement

The authors declare that the research was conducted in the absence of any commercial or financial relationships that could be construed as a potential conflict of interest.

## Footnotes

1 In Experiment 1, there was no accuracy-confidence correlation although there was clear non-linear trend in confidence that matched with non-linear trend in accuracy. In Experiment 2, there was positive accuracy-FOW correlation although there was no evidence for non-linear trend in FOW judgments.
