# Supplementary material for: Concurrent Dynamics of Category Learning and Metacognitive Judgments
Source: Front Psychol. 2016 Sep 27;7:1473. doi: 10.3389/fpsyg.2016.01473 (PMC5037202; doi:10.3389/fpsyg.2016.01473)
Supplement: Supplementary file 1 [file Data_Sheet_1.DOCX]

Supplemental Material

1. Trial-by-trial Analysis

The dynamics of category learning can be further decomposed at the trial level, as shown by Doyle and Hourian (2015). They examined whether a correct trial is more likely to be followed by a correct or incorrect trial. In the same manner, they analyzed likelihoods for incorrect trials. Doyle and Hourian (2015) predicted that participants learn more from successful trials rather than unsuccessful trials. Therefore, they predicted that a correct trial is more likely to be followed by a correct rather than an incorrect trial. At the same time, a correct or incorrect trial should be equally likely to occur after an incorrect trial, creating a so-called direction selectivity effect. Indeed, the data observed supported this prediction. Furthermore, an analysis of CLJs showed that the largest increase in CLJs is observed when a correct trial occurred after an incorrect trial.

Experiment 1

Following Doyle and Hourian (2015), we sorted data from each trial into four bins depending on the accuracy of the preceding trial on the same exemplar. Therefore, the correct-correct bin contained the number of times that a correct trial was followed by a correct trial, and the correct-incorrect bin contained the number of times that a correct trial was followed by an incorrect trial. The incorrect-correct and incorrect-incorrect bins were defined in the same way. These bins were computed separately for each type of task.

**It should be noted that making of statistical inferences based on such dataset is limited due to the fact that accuracy was above chance in all tasks and the sample size in estimating the incorrect-correct bin was necessarily smaller than in the correct-correct bin. In particular, we avoided direct comparisons of conditional probabilities between all four bins in each task. Instead, we performed a series of nonparametric tests to separately analyze differences in a) probability of correct or incorrect response occurring after a correct response, and b) probability of correct or incorrect response occurring after an incorrect response.**

A nonparametric Wilcoxon’s signed rank test was used to examine the differences in the probability of making correct (incorrect) classifications after a correct or incorrect classification. Descriptive data for trial-by-trial analysis is shown in Table S1. In task I, correct-correct was different from correct-incorrect, *Z* = 5.84, *p <* .01, while incorrect-correct also differed from incorrect-incorrect, *Z =* 5.14, *p <* .01. In the same way, in task II, correct-correct differed from correct-incorrect, *Z =* 5.75, *p <* .01, and incorrect-correct differed from incorrect-incorrect, *Z =* 2.40, *p <* .05. In task III, correct-correct differed from correct-incorrect, *Z =* 5.78, *p <* .01, but there were no differences between incorrect-correct and incorrect-incorrect, *Z =* 0.62, *p >* .05. This analysis revealed that only in task III is there an asymmetry, as predicted by Doyle and Hourian (2015); that is, correct trials were more likely to be followed by a correct response than by an incorrect response. However, incorrect trials were equally likely to be followed by correct or incorrect responses. In contrast, in tasks I and II, participants learned equally well from correct and incorrect trials, suggesting that errors and failures were equally informative in solving the task. Finally, we note that it was not possible to perform the same analysis on confidence judgments due to the large number of missing cells.

Table S1. Trial-by-trial accuracy contingencies in Experiments 1 & 2

| Trial Type | Correct - correct | Correct - incorrect | Incorrect - correct | Incorrect - incorrect |
| --- | --- | --- | --- | --- |
| *Experiment 1* |  |  |  |  |
| Task type I | .82 (.02) | .03 (.01) | .12 (.01) | .03 (.01) |
| Task type II | .57 (.02) | .10 (.01) | .18 (.01) | .14 (.01) |
| Task type III | .56 (.02) | .13 (.01) | .15 (.01) | .16 (.01) |
| *Experiment 2* |  |  |  |  |
| Task type I | .81 (.03) | .05 (.01) | .12 (.01) | .02 (.01) |
| Task type II | .52 (.03) | .13 (.01) | .20 (.01) | .16 (.01) |
| Task type III | .47 (.03) | .16 (.01) | .18 (.01) | .19 (.01) |

Note: Numbers in parentheses are the standard error of the mean.

Experiment 2

As in Experiment 1, we sorted the data from each trial into four bins (correct-correct, correct-incorrect, incorrect-correct, and incorrect-incorrect) depending on the accuracy of the preceding trial on the same exemplar. Therefore, the correct-correct bin contains the number of times that a correct trial was followed by a correct trial. These bins were computed separately for each type of task. Wilcoxon’s signed rank test was computed to examine the differences in the probability of making a correct (incorrect) classification after a correct or incorrect classification of the same item in the previous block. Descriptive data are shown in Table S1. In task I, correct-correct was different from correct-incorrect, *Z =* 5.37, *p <* .01, and incorrect-correct differed from incorrect-incorrect, *Z =* 5.23, *p <* .01. In task II, correct-correct differed from correct-incorrect, *Z =* 5.15, *p <* .01, and incorrect-correct differed from incorrect-incorrect, *Z =* 2.55, *p <* .05. In task III, correct-correct differed from correct-incorrect, *Z =* 5.22, *p <* .01, but there were no differences between incorrect-correct and incorrect-incorrect, *Z =* 1.08, *p >* .05. These data replicate findings from Experiment 1, suggesting that in tasks I and II, participants learn from successful trials as well as from error trials. Only task III revealed more learning from successes than from failures, as predicted by Doyle and Hourian (2015). It was not possible to do the same analysis on FOWs because they are given for a set of exemplars and not for each exemplar separately.

Discussion

We performed a trial-by-trial analysis of changes in accuracy similar to Doyle and Hourian (2015) and found partial support for their direction selectivity effect. In type I and II tasks, a correct trial is more likely to be followed by a correct rather than an incorrect trial. However, after an incorrect trial, a correct trial is also more likely to occur than an incorrect trial, suggesting that participants learn equally well from successful trials and from errors. This is expected to occur if participants generate and test hypotheses about the underlying rule. In this case, negative feedback is equally as informative as positive feedback because there are only two mutually exclusive categories. However, direction selectivity was observed in type III task in both experiments. Type III task is the most difficult to master, and it is possible that participants reverted to the strategy of exemplar memorization instead of hypothesis testing. Unfortunately, we did not ask participants the strategy they employed during category learning, so we cannot be sure whether there is direct evidence of a strategy shift between tasks. Further research is needed using strategy reports to address this issue (Little & McDaniel, 2015; Wahlheim et al., 2016). Nevertheless, our data suggests that when the task could be solved by formulating simple rules, participants learned from successes as well as from failures.

2. Trend Analyses

Table S2. Trend Analysis with all participants in Experiment 1

|  |  | Learning Task | | | | | | | | | | | |
| --- | --- | --- | --- | --- | --- | --- | --- | --- | --- | --- | --- | --- | --- |
|  |  |  | | Type I | |  | | Type II | |  | | Type III | |
| Trend |  |  | *F*(1, 42) | | *p* |  | *F*(1, 42) | | *p* |  | *F*(1, 42) | | *p* |
| Linear | ACC |  | **87.20** | | < .001 |  | **81.76** | | < .001 |  | **38.13** | | < .001 |
|  | CONF |  | **126.08** | | < .001 |  | **62.03** | | < .001 |  | **23.27** | | < .001 |
|  | RT |  | **118.27** | | <.001 |  | 3.30 | | > .50 |  | 6.55 | | .142 |
|  |  |  |  | |  |  |  | |  |  |  | |  |
| Quadratic | ACC |  | 3.52 | | .203 |  | 1.06 | | > .50 |  | 4.67 | | .146 |
|  | CONF |  | 3.64 | | .384 |  | < 1 | | > .50 |  | < 1 | | > .50 |
|  | RT |  | 2.73 | | > .50 |  | < 1 | | > .50 |  | < 1 | | > .50 |
|  |  |  |  | |  |  |  | |  |  |  | |  |
| Cubic | ACC |  | **17.43** | | .001 |  | **12.54** | | .006 |  | **13.79** | | .004 |
|  | CONF |  | **11.98** | | .011 |  | 2.07 | | > .50 |  | < 1 | | > .50 |
|  | RT |  | **13.97** | | .006 |  | < 1 | | > .50 |  | 1.46 | | > .50 |
|  |  |  |  | |  |  |  | |  |  |  | |  |
| Quartic | ACC |  | **28.08** | | < .001 |  | **7.51** | | .045 |  | < 1 | | > .50 |
|  | CONF |  | **10.54** | | .018 |  | 3.90 | | .384 |  | < 1 | | > .50 |
|  | RT |  | < 1 | | > .50 |  | < 1 | | > .50 |  | < 1 | | > .50 |

*Note*. Significant effects are highlighted by bold typeface. ACC, accuracy; CONF, confidence; RT, response times.

Table S3. Trend Analysis with successful participants in Experiment 1

|  |  | Learning Task | | | | | | | | | | | |
| --- | --- | --- | --- | --- | --- | --- | --- | --- | --- | --- | --- | --- | --- |
|  |  |  | | Type I | |  | | Type II | |  | | Type III | |
| Trend |  |  | *F*(1, 21) | | *p* |  | *F*(1, 21) | | *p* |  | *F*(1, 21) | | *p* |
| Linear | CONF |  | **91.86** | | < .001 |  | **68.90** | | < .001 |  | **38.13** | | < .001 |
|  | RT |  | **88.80** | | < .001 |  | 8.29 | | .090 |  | 7.63 | | .105 |
|  |  |  |  | |  |  |  | |  |  |  | |  |
| Quadratic | CONF |  | 6.75 | | .118 |  | < 1 | | > .50 |  | 2.96 | | > .50 |
|  | RT |  | 3.02 | | > .50 |  | 1.08 | | > .50 |  | < 1 | | > .50 |
|  |  |  |  | |  |  |  | |  |  |  | |  |
| Cubic | CONF |  | **16.05** | | .006 |  | 2.43 | | > .50 |  | < 1 | | > .50 |
|  | RT |  | **9.67** | | .058 |  | < 1 | | > .50 |  | < 1 | | > .50 |
|  |  |  |  | |  |  |  | |  |  |  | |  |
| Quartic | CONF |  | 1.47 | | > .50 |  | **9.23** | | .050 |  | < 1 | | > .50 |
|  | RT |  | < 1 | | > .50 |  | 2.65 | | > .50 |  | < 1 | | > .50 |

*Note*. Significant effects are highlighted by bold typeface. CONF, confidence; RT, response times.

Table S4. Trend Analysis with all participants in Experiment 2

|  |  | Learning Task | | | | | | | | | | | |
| --- | --- | --- | --- | --- | --- | --- | --- | --- | --- | --- | --- | --- | --- |
|  |  |  | | Type I | |  | | Type II | |  | | Type III | |
| Trend |  |  | *F*(1, 34) | | *p* |  | *F*(1, 34) | | *p* |  | *F*(1, 34) | | *p* |
| Linear | ACC |  | **74.79** | | < .001 |  | **44.48** | | < .001 |  | **27.14** | | < .001 |
|  | CONF |  | **25.62** | | < .001 |  | **41.17** | | < .001 |  | **30.49** | | < .001 |
|  | RT |  | **42.46** | | <.001 |  | 4.63 | | .309 |  | **13.44** | | .009 |
|  |  |  |  | |  |  |  | |  |  |  | |  |
| Quadratic | ACC |  | < 1 | | > .50 |  | < 1 | | > .50 |  | < 1 | | > .50 |
|  | CONF |  | < 1 | | > .50 |  | < 1 | | > .50 |  | < 1 | | > .50 |
|  | RT |  | 2.31 | | > .50 |  | 4.90 | | .303 |  | 2.26 | | > .50 |
|  |  |  |  | |  |  |  | |  |  |  | |  |
| Cubic | ACC |  | **10.11** | | .022 |  | **16.14** | | .002 |  | **8.48** | | .038 |
|  | CONF |  | 8.14 | | .059 |  | **14.03** | | .006 |  | 1.71 | | > .50 |
|  | RT |  | 1.93 | | > .50 |  | 2.09 | | > .50 |  | < 1 | | > .50 |
|  |  |  |  | |  |  |  | |  |  |  | |  |
| Quartic | ACC |  | **38.25** | | < .001 |  | 1.80 | | > .50 |  | 1.77 | | > .50 |
|  | CONF |  | < 1 | | > .50 |  | 2.68 | | > .50 |  | 5.98 | | .139 |
|  | RT |  | **9.76** | | .036 |  | < 1 | | > .50 |  | < 1 | | > .50 |

*Note*. Significant effects are highlighted by bold typeface. ACC, accuracy; CONF, confidence; RT, response times.

Table S5. Trend Analysis with successful participants in Experiment 2

|  |  | Learning Task | | | | | | | | | | | |
| --- | --- | --- | --- | --- | --- | --- | --- | --- | --- | --- | --- | --- | --- |
|  |  |  | | Type I | |  | | Type II | |  | | Type III | |
| Trend |  |  | *F*(1, 21) | | *p* |  | *F*(1, 21) | | *p* |  | *F*(1, 21) | | *p* |
| Linear | CONF |  | **17.99** | | .004 |  | **53.58** | | < .001 |  | **110.35** | | < .001 |
|  | RT |  | **37.06** | | < .001 |  | 6.32 | | .161 |  | **18.43** | | .004 |
|  |  |  |  | |  |  |  | |  |  |  | |  |
| Quadratic | CONF |  | < 1 | | > .50 |  | < 1 | | > .50 |  | < 1 | | > .50 |
|  | RT |  | 1.26 | | > .50 |  | 3.31 | | > .50 |  | **13.52** | | .014 |
|  |  |  |  | |  |  |  | |  |  |  | |  |
| Cubic | CONF |  | 4.84 | | .274 |  | **9.56** | | .044 |  | 1.27 | | > .50 |
|  | RT |  | 3.09 | | > .50 |  | 1.67 | | > .50 |  | < 1 | | > .50 |
|  |  |  |  | |  |  |  | |  |  |  | |  |
| Quartic | CONF |  | 2.85 | | > .50 |  | < 1 | | > .50 |  | **16.02** | | .006 |
|  | RT |  | **9.56** | | .050 |  | 1.21 | | > .50 |  | 1.82 | | > .50 |

*Note*. Significant effects are highlighted by bold typeface. CONF, confidence; RT, response times.
